# miRNA regulation in brain tissue space: the 3′UTR perspective

**DOI:** 10.1261/rna.080850.125

**Published:** 2026-04

**Authors:** Denise Aigner, Florian Bartsch, Poojashree Bhaskar, Lisa Emmenegger, Nikos Karaiskos, Nikolaus Rajewsky, Agnieszka Rybak-Wolf

**Affiliations:** 1 Laboratory for Systems Biology of Regulatory Elements, Berlin Institute for Medical Systems Biology (BIMSB), Max-Delbrück-Centrum for Molecular Medicine in the Helmholtz Association (MDC), 10115 Berlin, Germany; 2 Humboldt-Universität zu Berlin, 10117 Berlin, Germany; 3 Department of Pediatric Oncology and Hematology, Charité-Universitätsmedizin Berlin, 13353 Berlin, Germany; 4 Charité-Universitätsmedizin Berlin, 10117 Berlin, Germany; 5 German Center for Cardiovascular Research (DZHK), 10785 Berlin, Germany; 6 NeuroCure Cluster of Excellence, 10117 Berlin, Germany; 7 German Cancer Consortium (DKTK), 69120 Heidelberg, Germany; 8 National Center for Tumor Diseases (NCT), 69120 Heidelberg, Germany; 9 Organoid Platform, Berlin Institute for Medical Systems Biology, Max Delbrück Center for Molecular Medicine in the Helmholtz Association (MDC), 10115 Berlin, Germany

**Keywords:** microRNA, 3'UTR isoforms, brain, spatial quantification, sequencing

## Abstract

MicroRNAs (miRNAs) are key regulators of gene expression in both health and disease. Their expression and regulatory functions are highly complex and spatiotemporally organized within tissues. In recent years, spatial transcriptomics has made significant progress in quantifying RNA expression at subcellular resolution in tissue sections. However, no current method can quantify miRNAs and their target 3′ untranslated regions (3′UTRs) in space simultaneously. Furthermore, although 3′UTRs harbor critical miRNA target sites, 3′UTR isoform variation in space is largely unexplored. In this review, we discuss the role of miRNA-mediated regulation. We focus on neurodevelopment and neuronal function, where miRNAs and 3′UTRs have particularly complex and important functions. We summarize current experimental and computational approaches for spatial quantification of miRNAs and 3′UTRs, highlight existing challenges, and propose strategies for future research.

## miRNAs AS SYSTEMS-LEVEL REGULATORS

### Origin and biogenesis of miRNAs

Posttranscriptional regulation shapes gene expression by controlling mRNA abundance, stability, localization, and translation. Among its principal mediators are microRNAs (miRNAs), which are small noncoding RNAs, 20–24 nt in length. The discovery of miRNAs in the roundworm *Caenorhabditis elegans* by Victor Ambros and Gary Ruvkun unveiled a novel layer of complexity in posttranscriptional gene regulation, an achievement recognized by the 2024 Nobel Prize in Physiology or Medicine (Ruvkun and Giusto 1989; Lee et al. 1993; Wightman et al. 1993).

**FIGURE 1.**
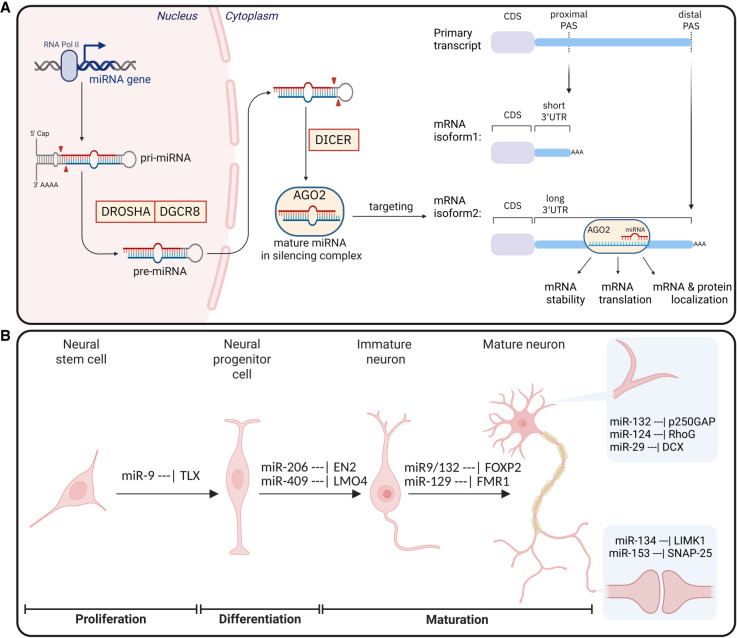
Overview of miRNA and 3′UTR biogenesis and their interaction during neurodevelopment and neuronal function. (*A*) Biogenesis of miRNAs mediated by DROSHA, DGCR8 and DICER until the final mature miRNA is loaded via AGO2 for targeting of mRNA isoforms influencing stability, translation or protein localization of the target mRNA. (*B*) Examples of miRNAs and their role in neural development. miRNAs can be very specific for certain subcellular compartments.

Most miRNAs are transcribed from noncoding regions, or introns of their host genes, with a few originating from protein-coding exons (Rodriguez et al. 2004; Baskerville and Bartel 2005; Kim and Kim 2007). In the canonical pathway, RNA polymerase II transcribes primary miRNAs (pri-miRNAs), which fold into hairpin structures (Cai et al. 2004; Lee et al. 2004). Those are first processed by DROSHA-DGCR8 (DiGeorge syndrome critical region 8) into pre-miRNAs (Denli et al. 2004; Gregory et al. 2004; Han et al. 2004; Landthaler et al. 2004) and then by DICER into ∼22 nt duplexes (Grishok et al. 2001; Hutvágner et al. 2001). One miRNA strand is selectively retained in Argonaute (AGO) to form the effector complex (Khvorova et al. 2003; Schwarz et al. 2003), targeting specific transcripts based on the complementarity of its seed sequence (positions 2–8 from the miRNA 5′ end) (Fig. 1A, left; reviewed by Bartel 2009; Jonas and Izaurralde 2015). However, some miRNAs, known as mirtrons, originate from short intronic sequences (Okamura et al. 2007; Ruby et al. 2007), bypassing DROSHA processing and being instead directly cleaved by DICER (Berezikov et al. 2007). This flexibility in miRNA biogenesis allows cells to fine-tune gene expression under different contexts of time and space.

### Consequences of miRNA evolution in neural systems

miRNAs are ancient molecules that emerged in multicellular organisms and expanded during animal evolution, in particular in vertebrates (Vertebrata to Simiiformes, Fromm et al. 2020; Clarke et al. 2025). Strikingly, cephalopods, which independently from vertebrates developed a complex nervous system, also exhibit a similar expansion of miRNAs, with novel miRNAs particularly enriched in neuronal tissues (Zolotarov et al. 2022). This suggests a correlation between increased neural complexity and miRNA repertoire expansion (Heimberg et al. 2008; Zolotarov et al. 2022). Moreover, primate-specific miRNAs might contribute to species-specific brain traits, influencing neuronal maturation and function (Hu et al. 2012; Lopez et al. 2014; Prodromidou et al. 2020).

This overarching evolutionary trend reflects the diverse repertoire of miRNAs, especially in brain tissues (Landgraf et al. 2007; Ludwig et al. 2016). Here, miRNAs modulate neuron development and function (Rajman and Schratt 2017). Moreover, miRNA-dependent regulatory networks exhibit strong evolutionary conservation across species (Grün et al. 2005). In all analyzed organisms, including mice, constitutive knockouts of *Dicer* or *Drosha* cause severe developmental defects and ultimately lead to embryonic lethality (reviewed in detail by Alberti and Cochella 2017). For instance, loss of Drosha impairs gastrulation in sea urchins (Song et al. 2012) and organogenesis in *C. elegans* (Dexheimer et al. 2020), while loss of Dicer affects brain morphogenesis in zebrafish (Giraldez et al. 2005). In all these cases, the defects could be partially rescued by one or two miRNA families, suggesting that, at the onset of embryogenesis, relatively few, broadly and abundantly expressed miRNAs are essential. As development progresses and cells specialize, miRNAs acquire increasingly high cell-type specificity, acting as precise spatial and temporal regulators of gene expression (Alberti et al. 2018; Rahman et al. 2020). In multicellular organisms, the lack of approaches with cellular resolution has long obscured the detection of highly abundant miRNAs expressed in only a few cells. Notably, some miRNAs were found to be highly enriched in specific cell types, suggesting that even those with low broad abundance across tissue could play critical roles when expressed in a highly cell-type-specific manner (Alberti and Cochella 2017). For example, the *C. elegans* miRNA lsy-6 is expressed and functionally required in just a single neuron in the entire adult animal (Cochella and Hobert 2012). Taken together, miRNAs have coevolved with increasing organismal and neural complexity and, as key regulators of gene expression, contribute to proper developmental progression and precise cell-type specification.

### miRNAs at work

In animals, miRNAs target the 3′ untranslated regions (3′UTRs) of mRNAs through sequence-specific, mostly partial, base-pairing (reviewed by Bartel 2009). mRNA destabilization driven by deadenylation and decapping is the common outcome. However, other effects like translational repression and target-directed miRNA decay (TDMD) can also occur (Filipowicz et al. 2008; Selbach et al. 2008; Cazalla et al. 2010; De La Mata et al. 2015; Jonas and Izaurralde 2015; Bartel 2018; Kleaveland et al. 2018). In particular, TDMD can arise when extensive sequence complementarity between the miRNA and its target RNA leads to the degradation of the miRNA itself. The Bartel group showed that TDMD can strongly modulate miRNA expression in neural systems. For example, loss of a single binding site that causes TDMD has been shown to induce up to 10-fold increase in miRNA expression (Kleaveland et al. 2018).

A single miRNA can bind to numerous functionally important targets (Lewis et al. 2003, 2005; Krek et al. 2005), which might result in distributed and subtle repression (Baek et al. 2008; Selbach et al. 2008), as evidenced primarily in cell lines. Such subtle effects stabilize developmental transitions, like the shift from progenitor cells to neurons (Hornstein and Shomron 2006; Zhao et al. 2009; Yoo et al. 2011; Ebert and Sharp 2012; Schmiedel et al. 2015). However, when multiple miRNAs bind cooperatively to the same 3′UTR in proximity, they can achieve even stronger repression (Krek et al. 2005; Sætrom et al. 2007; Mukherji et al. 2011; Denzler et al. 2016). This combinatorial approach allows miRNAs to adjust pathways with precision and context-specificity, which is especially crucial for precise spatiotemporal gene regulation in polarized cells such as neurons (Wayman et al. 2008; Impey et al. 2010; Cohen et al. 2011; Tan et al. 2013).

However, the consequences of miRNAs binding to their targets are more nuanced. Competition for binding to their targets, as well as the presence of other competing endogenous RNAs (ceRNAs) with the same miRNA binding sites, further complicates the regulatory landscape (Jens and Rajewsky 2015). ceRNAs can sequester miRNAs, dampening their interactions with 3′UTR targets. One such interaction in the brain is between miR-7 and a circular RNA called CDR1as, which has over ∼74 binding sites for miR-7 in humans. Studies revealed that sequestering of miR-7 by CDR1as has downstream functional consequences, further shaping the miR-7-dependent regulation (Hansen et al. 2013; Memczak et al. 2013).

Therefore, through their various interactions, miRNA repertoires might provide the brain with scalable tools for robust, temporally precise, and compartment-specific control of gene expression. Yet, miRNAs never act in isolation: Their impact depends heavily on their targets—3′UTRs. Thus, to interpret miRNA function in the brain, we must first decode the properties of their targets.

## 3′UTRs

### 3′UTR dynamics

3′UTRs are noncoding regions located downstream from the coding sequence and upstream of the poly(A) tail. They encode crucial regulatory information, as they contain not only binding sites for miRNAs but are also targeted by a repertoire of >1000 RNA-binding proteins (RBPs); ENCODE biochemically mapped in vivo binding sites for hundreds of RBPs across the transcriptome in human cells (Van Nostrand et al. 2020). Consequently, 3′UTR isoforms influence different regulatory functions (reviewed by Mayr 2016, 2017). Through alternative polyadenylation (APA), nascent mRNA transcripts with different poly(A) sites (PAS) generate multiple isoforms of varying 3′UTR length (Fig. 1A, right; Licatalosi and Darnell 2010; Tian and Manley 2017). Proximal PAS selection yields shorter 3′UTRs that can evade regulatory control, while distal-site usage produces longer 3′UTRs enriched in *cis*-regulatory elements. It was initially proposed that such modulation of 3′UTR length introduced *cis*-regulatory elements, which were thought to influence mRNA stability. Among these elements were miRNA-binding sites that could confer stronger posttranscriptional repression. Moreover, it was later shown that the position of miRNA-binding sites further influenced the extent of repression, with binding sites located near both ends of the 3′UTRs showing stronger repression (Gaidatzis et al. 2007; Hoffman et al. 2016). Nevertheless, the model systems used for the experiments can shape the narrative. Mayr (2019) reviews how metabolic differences in primary cells and cell lines not only affect isoform diversity, but also the extent of miRNA-dependent repression (Mayr 2019). Conversely, shorter isoforms, by losing these miRNA-binding sites, evade such repression, resulting in high mRNA stability and producing up to 10-fold higher protein levels (Lee and Dutta 2007; Mayr et al. 2007; Mayr and Bartel 2009). 3′UTR shortening was identified as a mechanism for modulating gene expression in diverse biological contexts, including T-cell activation (Sandberg et al. 2008) and embryonic development (Ji et al. 2009).

In this manner, 3′UTR isoform selection seems to be influenced by multiple factors and is cell-type- and context-specific, expanding the posttranscriptional regulatory diversity.

### Regulation of 3′UTR isoform diversity

3′UTRs, like miRNAs, exhibit distinct evolutionary dynamics. While coding regions and 5′UTRs of mRNAs have remained relatively constant in size across species, their 3′UTRs have lengthened considerably, from ∼140 nt in worms to >1200 nt in humans, especially in neurons (Sood et al. 2006; Mayr 2016, 2017). This elongation enabled more interactions with miRNAs and RBPs, facilitating precise regulatory control over genes (Mayr and Bartel 2009; Ebert and Sharp 2012; Miura et al. 2013). Consistent with this, more than 70% of neuron-enriched genes express multiple long 3′UTR isoforms. However, 3′UTR lengthening seems to be tissue- and cell-type-specific, as proliferating and cancer cells favor shorter 3′UTR isoforms, possibly to evade mRNA repression (Sandberg et al. 2008; Ji et al. 2009; Mayr and Bartel 2009; Derti et al. 2012; Miura et al. 2013; Tushev et al. 2018). It has been shown that the enrichment of 3′UTR shortening correlates with poorer prognosis in various tumors (Rosenwald et al. 2003; Wiestner et al. 2007; Huang et al. 2018; Sagredo et al. 2018; Jia et al. 2022), with 3′UTR lengthening described as a pro-senescence and anticancer mechanism in some cases (Chen et al. 2018). While the mechanisms underlying the preferential use of proximal APA in tumor cells remain poorly understood, recent CRISPR-based screens targeting endogenous APA are beginning to shed light on this process (Gabel et al. 2024). However, understanding 3′UTR length alone will not answer all questions as subsequent studies have suggested that its influence on mRNA stability and protein levels may be less pronounced than originally thought (Spies et al. 2013; Gruber et al. 2014). A striking example is the Pten tumor suppressor gene, for which longer 3′UTR isoforms were found to be highly stable, supporting robust protein production and contributing most strongly to Pten signaling (Thivierge et al. 2018). Additionally, in certain cell lines, 3′UTR length variation appears to play only a minor role in the regulation of mRNA stability (Spies et al. 2013; Gruber et al. 2014).

Nevertheless, in the brain, the trend of long 3′UTR isoforms seems to be conserved across species, which might be promoted by distal PAS selection by neuron-specific RBPs like ELAV/Hu (Hilgers et al. 2012; Miura et al. 2013). Recently, it was revealed that transcriptional start site selection may influence PAS selection in brains from flies to humans (Legnini et al. 2019; Alfonso-Gonzalez et al. 2023).

### Roles of neuronal 3′UTRs

The increase of neuronal 3′UTR isoforms is driven by the functional needs of such highly polarized cells. It has been proposed that *cis*-regulatory elements in 3′UTRs can act as zip codes for some mRNAs like β*-actin* and *Camk2A* (Kislauskis et al. 1994; Mayford et al. 1996), guiding their subcellular localization. However, it has also been suggested that mRNA stability might be the prime signal controlling the “outreach” of mRNAs in neurites (Loedige et al. 2023). Nevertheless, not all 3′UTR isoforms contribute to localization in the same manner. Inconsistent findings have been reported regarding the stability and dendritic localization of the long versus short isoforms of *bdnf* (An et al. 2008; Will et al. 2013), highlighting how such dynamics are context-dependent and may not be captured efficiently with single-plex assays. This underscores how gene-specific behaviors complicate generalization and necessitate unbiased multiplex assays to resolve 3′UTR isoforms and the miRNAs they are targeted by.

## FROM STEMNESS TO SYNAPSE: miRNAs–3′UTR INTERACTIONS IN THE BRAIN

miRNAs–3′UTR target interactions are crucial for neurodevelopment and neuronal function (Fig. 1B). During early development, miRNA and their targets regulate decisions, balancing neural stem cell (NSC) proliferation and differentiation. One such case is the proposed feedback loop between miR-9/TLX, with the disruption of miR-9 resulting in severe cortical malformations (Zhao et al. 2009; Shibata et al. 2011). Other miRNAs, such as miR-20a/20b and miR-23a, work potentially in a feedback loop with cyclin-D1 acting as temporal regulators of cortical neurogenesis (Ghosh et al. 2014).

As neural progenitors mature into specialized neurons, these interactions further define their identities. Some miRNAs, such as miR-206, act as key regulators by suppressing essential TFs like *En2*, regulating Purkinje cell fate (Zolboot et al. 2025). In comparison, others exhibit region-specific interactions while shaping the same cell-type specification. For example, while miR-409 and *Lmo4* interaction may shape motor neuron identity in the cortex (Diaz et al. 2020), miR-218 regulation of the *Isl1–Lhx3* complex influences the same identity in the developing spinal cord.

Accordingly, miR-218 loss is associated with hallmark features of motor neuron disorders (Amin et al. 2015; Thiebes et al. 2015).

Beyond neuronal fate specification, miRNAs are integral to temporally and spatially regulated processes such as neuronal migration and synaptic activity. For example, miR-9, miR-132, and miR-129 target *Foxp2* (miR-9 and miR-132) and *Fmr1* (miR-129), both autism-associated genes, illustrating the importance of timely miRNA-mediated repression for proper cortical migration (Clovis et al. 2012; Wu et al. 2019). Furthermore, in mature neurons, miRNAs coordinate processes like dendritic growth, synaptogenesis, and neurotransmission. Some miRNAs can remodel dendritic architecture by indirectly targeting the cytoskeletal machinery elements, such as miR-124:*RhoG* and miR-29a:*Dcx* interactions (Franke et al. 2012; Li et al. 2014), influencing neuronal circuits.

miRNAs also play a direct role in synaptic compartments, regulating neuronal activity. This regulation is spatially controlled to a level that specific interactions occur at particular compartments. At postsynaptic sites, miR-134 regulates neuronal excitability by repressing *Limk1* (Schratt et al. 2006). Presynaptically, miR-153 regulates vesicle release by targeting *Snap-25* (Wei et al. 2013), and miR-128 constrains intrinsic excitability through an ERK2-centered network and ion-channel regulators, where its loss can lead to fatal epilepsy (Tan et al. 2013). Conversely, neuronal activity can also affect miRNA regulation. For example, the CREB pathway regulates miR-132, which in turn influences its target, p250GAP. This interaction affects dendrite morphogenesis and influences structural and functional plasticity (Vo et al. 2005; Wayman et al. 2008). Notably, miR-132 and miR-134 have been implicated in the progression of seizures in epilepsy, suggesting an underlying mechanism (Jimenez-Mateos and Henshall 2013).

Collectively, these studies illuminate how miRNA–3′UTR interactions regulate neural development and maturation, guiding progenitor decisions, refining neuronal identities, regulating migration, and fine-tuning synaptic dynamics. The outcome of any particular miRNA activity is thus heavily influenced by context, depending on factors like cell identity, brain region, subcellular location, and the range of 3′UTR isoforms expressed.

## ONE miRNA, MANY FUNCTIONS: IMPLICATIONS IN PHYSIOLOGY AND DISEASE

Regulation by miRNAs is often context-dependent. For instance, the same miRNA can silence stemness in one context, while modulating synaptic function in another, whether under physiological or pathological conditions. The let-7 family illustrates the most famous context-dependent functional diversity: In NSCs, let-7 represses *SOX2* and *LIN28*, orchestrating the transition from proliferation to differentiation (Rybak et al. 2008; Cimadamore et al. 2013), whereas in mature neurons, it modulates neurite outgrowth through actin-cytoskeletal targets (McGowan et al. 2018). Additionally, let-7 expression increases with aging and has been proposed to play a role in Alzheimer's disease by regulating amyloid precursor protein-like (APL-1) gene through lin-41 (Niwa et al. 2008; Shamsuzzama et al. 2016). In contrast, low let-7 levels lead to upregulation of its target E2F1, promoting dopaminergic neuron death, a phenotype linked to Parkinson's disease (Gehrke et al. 2010).

Similarly, miR-7 demonstrates remarkable functional versatility. During early neuronal specification, it interacts with TFs such as *Olig2* and *Pax6*, helping shape dopaminergic lineages and cortical patterning (de Chevigny et al. 2012; Pollock et al. 2014). Later, in forebrain neurons, miR-7 fine-tunes glutamatergic transmission by modulating glutamatergic receptor expression (Cerda-Jara et al. 2024; Scoyni et al. 2024). And in mature dopaminergic neurons, miR-7 directly regulates α-*synuclein*, central to Parkinson's disease pathogenesis (Junn et al. 2009; Doxakis 2010).

Even miRNAs traditionally associated with one role reveal unexpected diversity. miR-133b is classically viewed as a regulator of midbrain dopaminergic neuron development through *Pitx3* (Kim et al. 2007). Yet, it is also expressed in a distinct population of interneurons, where it engages an entirely different gene network (He et al. 2012). Adding to this, dysregulation of miR-133b is also implicated in central poststroke pain in forebrain regions, emphasizing that miRNA functions are not only temporally but also spatially and cell type restricted (Guo et al. 2022).

Together, these examples demonstrate a key principle: miRNA regulation is not static but is highly context-dependent, influenced by factors such as cell type, brain region, subcellular compartment, and the accessibility of target transcripts. Consequently, the same miRNA can drive developmental processes, stabilize neuronal morphology, or impact pathology, depending on the timing and location of its action. However, the examples mentioned primarily utilize luciferase reporter assays to test miRNA interactions with 3′UTRs, or employ antagomiRs and mimics to infer downstream effects. These methods could be potentially influenced by external factors. The ideal approach would involve mutating the endogenous binding sites within the 3′UTR targets to establish direct dependency (Lai 2004; Labi et al. 2019; Froehlich and Rajewsky 2023), but such experiments are rare due to their complexity and tedious nature. Therefore, to advance from merely correlational relationships to a more mechanistic understanding, the field would benefit from methods that can simultaneously detect miRNAs and their 3′UTR targets within their specific cellular and subcellular contexts in situ.

## FROM NORTHERN BLOTS TO SPATIAL ISOFORMS: THE EVOLVING TOOLKIT TO CAPTURE miRNAs AND 3′UTRs

**FIGURE 2.**
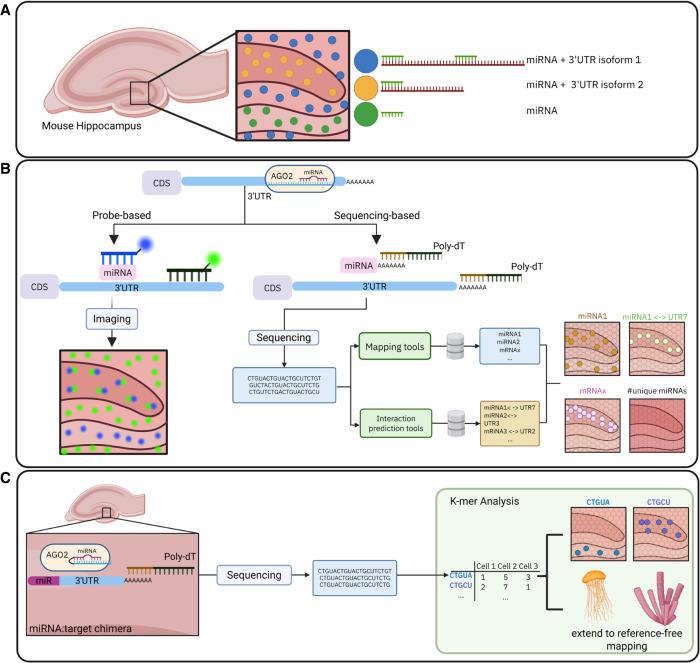
Methodologies for detecting the spatial context of miRNA-UTR interactions. (*A*) Conceptual illustration of miRNA abundance in the mouse hippocampus and interactions with different 3′UTR isoforms. (*B*) Overview of current state-of-the-art probe-based imaging (In situ hybridization) and sequencing-based (Poly-Adenylation) workflows. Sequencing-based methods rely on external databases (e.g., Human Genome, miRNA-target databases) and can identify spatial patterns of miRNAs, mRNAs and miRNA-3′UTR interactions in high-throughput. (*C*) Potential future workflows for miRNA-UTR spatial analysis include, on the experimental side, ligation-based approaches to construct miRNA:target chimeras directly on tissue capture arrays. On the computational side, *k*-mer analysis could be used to detect spatial patterns of sequences without relying on a reference genome, even enabling cross-species analysis.

The previous section highlighted the crucial role that miRNAs and their 3′UTR targets play in gene regulation. To fully understand this relationship, methods capable of simultaneous detection of miRNAs and their corresponding 3′UTR isoforms in situ, at single-cell and subcellular resolutions, are necessary (Fig. 2A). Despite recent advancements, significant challenges remain. This section reviews current progress and outlines ongoing obstacles in achieving precise spatial quantification of miRNAs and 3′UTR isoforms.

### Breaking the bulk barrier: early attempts at spatial detection

The quest to measure miRNAs and 3′UTR isoforms has always been a challenge. miRNAs are short and sparse, making them difficult targets for detection. Initially, mature miRNA molecules were detected using well-known molecular assays like northern blot, qPCR and TaqMan assays (Chen et al. 2005; Lunn et al. 2008), and microarrays (Beuvink et al. 2007). While these methods offered the first glimpses of their expression, they relied on bulk tissue samples, which averaged signals, and resulted in the loss of critical cell type and spatial information (Raymond et al. 2005; Sood et al. 2006). The first real step toward spatial insight came with the development of in situ hybridization (ISH), a probe- and imaging-based technique offering high specificity and providing up to single-molecule resolution (Fig. 2B, left; Kloosterman et al. 2006; Lu and Tsourkas 2009; Haimovich and Gerst 2018). This method allowed the design of custom probes and fluorophore labeling to detect specific miRNA and 3′UTR isoform expression in situ and their intersection with protein networks. However, the lack of technical scalability and multiplexing limited its throughput (Urbanek et al. 2015; Zhuang et al. 2020). To overcome this, creative engineering approaches such as grid-based microwell systems (Nagarajan et al. 2020) and diamond nanoneedles (Wang et al. 2020) were developed. This improved multiplexing capacity and spatial resolution, but was limited by the number of fluorophores available. In parallel, in situ detection methods of 3′UTR isoforms also relied on ISH (Hilgers et al. 2011), and microarray-based strategies such as probe-level alternative transcript analysis (PLATA) (Sandberg et al. 2008; Ji et al. 2009). Nevertheless, similar to miRNA detection strategies, these methods faced challenges with resolution and throughput, as microarrays struggled with isoform diversity and scalability due to the limitations of probe design. Despite current advances, there is still a need for unbiased approaches to overcome these limitations.

### Short but complex: experimental and computational pitfalls in miRNA sequencing

Recognizing the bottlenecks of probe-based methods, unbiased spatial sequencing techniques emerged as promising, powerful alternatives, holding the potential to map miRNAs (Stickels et al. 2021; Schott et al. 2024; de Oliveira et al. 2025).

#### Experimental methods for miRNA investigation

miRNAs detection in bulk has already been done since 2005 and is often based on the ligation of adapters to both ends of the miRNAs (for review, see Benesova et al. 2021). However, routine quantification of miRNAs in single cells remains a challenge due to their small size and lack of poly(A) tail, which limits their capture using standard protocols. In standard poly(A) tail–capturing sequencing methods, miRNAs can only be indirectly captured in the form of the primary transcripts. An innovative solution for this challenge is the external polyadenylation of all RNA molecules, for example, in the vast transcriptome analysis of single cells by dA-tailing (Vasa-seq) and spatial total RNA-sequencing (STRS) method (Fig. 2B, right; Salmen et al. 2022; McKellar et al. 2023). However, an unwanted side effect of polyadenylating every molecule in a cell is the capture of highly abundant high ribosomal RNA (rRNA). To mitigate this rRNA contamination, Patho-DBit incorporated rRNA depletion, with a pixelated microfluidics system, achieving near single-cell resolution (10–25 µm) and compatibility with formalin-fixed, paraffin-embedded (FFPE) tissues (Bai et al. 2024). Additionally, given that most mature miRNAs are loaded in AGO, these miRNAs might not be accessible for 3′ end modifications such as poly(A) tailing (Wang et al. 2008; Schirle et al. 2014). RNA fragmentation during paraffin embedding could boost capture efficiency by releasing AGO-bound miRNA.

#### Computational methods for miRNA detection

While these strategies enhance miRNA capture in single-cell and spatial sequencing techniques, the difficulties extend well beyond the bench, leaving significant challenges for the computational analysis. One of those constraints lies in the alignment of miRNAs, where the high sensitivity to even single mismatches while mapping to a reference genome causes mapping errors and inflates false positives. This is exacerbated by the high similarity between miRNA families or isomiRs (miRNAs that differ only in a few nucleotides but target largely the same RNA transcripts). Since conventional aligners (e.g., STAR, HISAT2) are optimized for longer transcripts, they often require stringent settings or specialized small-RNA pipelines for miRNA mapping (Dobin et al. 2013; Kim et al. 2019). However, short-read aligners, such as Segemehl, improve this accuracy by accommodating subtle edits and indels (Hoffmann et al. 2009). Moreover, specialized pipelines like miRDeep2 enable quantitative detection of known miRNAs and discovery of novel miRNAs, thereby providing integrated miRNA profiling (Friedländer et al. 2012). Subsequent pipelines such as CAP-miRSeq reuse parts of miRDeep2 for mapping and are also a comprehensive solution for miRNA discovery, mapping, and annotation (Sun et al. 2014).

Further downstream expression analysis also has several caveats where standard methods, such as DESeq2, need to be used cautiously, especially for low-count miRNAs (Love et al. 2014). miRge3.0 aims to address these limitations by incorporating UMI deduplication, error modeling, and isomiR-aware quantification (Patil and Halushka 2021). Pushing this further ahead, graph-convolutional networks and small-RNA-specific normalization methods (Düren et al. 2022) continue to improve miRNA quantification accuracy. This highlights the need for dedicated computational pipelines, where each layer of analysis demands its own innovation to efficiently quantify small molecules. Moreover, these methods have to be directly compatible and adjusted to single-cell or spatial transcriptomics methods, where scalability, barcode/UMI handling, and spatial indexing are critical requirements and would require careful adjustments.

### Longer and not less complex: decoding 3′UTR isoform complexity

When studies began to explore 3′UTR regulation in vivo within a spatial context, for instance in *C. elegans*, it became evident that 3′UTR dynamics are tightly coupled to developmental and cellular organization. Early work by Mangone et al. in 2010 demonstrated that miRNAs coordinate widespread posttranscriptional regulation of mRNAs through their 3′UTRs in the *C. elegans* germline, revealing spatiotemporally restricted miRNA–target interactions (Mangone et al. 2010; Diag et al. 2018).

#### 3′UTR quantification from bulk sequencing

The process of analyzing 3′UTR isoforms in sequencing data is a multistep process that takes into account PAS usage and APA events to infer the presence and functionality of different 3′UTR isoforms. To fully decipher and dissect the diversity and functional specificity hidden within different 3′UTR isoforms, several sequencing-based approaches such as PAS-seq (Shepard et al. 2011), 3′READS/3′READS+ (Hoque et al. 2013; Zheng et al. 2016), and A-seq/A-seq2 (Martin et al. 2012, 2017) were developed to access 3′UTRs in cell lines. On the other hand, in a cross-comparison between *C. elegans* and human, 3P-seq showed that the 3′UTRs in *C. elegans* were not only shorter, but also exhibited a higher density for miRNA binding sites to compensate miRNA activity (Jan et al. 2011). To get more insight into a specific cell type, Gruber et al. coupled 3′ sequencing data and quantitative mass spectrometry to investigate T-cell activation. Even though they could only detect minor changes in 3′UTR shortening with no correlation to changed protein levels, this indicates and underlines that different 3′UTR isoforms might differ more between cell types than between activational changes in the same cell (Gruber et al. 2014).

#### 3′UTR isoform detection with single-cell resolution

To further dive into the cell type–specific expression pattern of different 3′UTR isoforms, the need for methods to move beyond bulk RNA-seq and decipher APA usage of multicellular organisms in single-cell resolution increased. As scRNA-seq methods coupled with short-read sequencing became more and more established, scientists leveraged the abundance of existing scRNA-seq data sets for 3′UTR isoform discovery with newly developed computational tools.

As most gene expression matrices already collapse 3′UTR isoform counts to reduce data sparsity, they typically lack detailed sequence-level information needed for APA analyses. Consequently, many computational tools directly leverage raw sequencing data (often directly from BAM/SAM files) rather than summarized count matrices. Tools such as scDAPA (Ye et al. 2020), SCINPAS (Moon et al. 2023), and SCAPE/SCAPE-APA (Zhou et al. 2022; Cheng et al. 2025) try to infer differential APA signatures from short-read scRNA-seq. These, among other tools, have been intensively reviewed by Fahmi et al. (2025). Despite the vast amount of available short-read scRNA-seq data, precise identification of PAS remains challenging because reads in 10x Genomics data often do not extend fully to the 3′ mRNA cleavage site (CS). To address this, scUTRquant built a CS atlas based on full-length transcriptome data (SMART-seq2 protocol) of mouse and human cell types. The intersection of this atlas with scRNA-seq data resulted in a variety of novel detected CS and demonstrated that 3′UTR isoform expression and length strongly differ between different cell types (Fansler et al. 2024).

To extend single-cell analyses into spatial contexts, spatial barcoding of cells is required. stAPAminer combines single-cell sequencing data and spatially barcoded cells to compute poly(A) site–spot matrices and relative usages of the distal poly(A) site. These are then projected in spatial context for downstream analysis of spatial patterns (Ji et al. 2023). More recently, integrative frameworks like spvAPA have extended these efforts to spatial transcriptomics, providing a method to identify region-specific 3′UTR isoforms and improving the discovery of cell subtypes by having BAM files as input. Here, APA events were inferred by identifying genome-wide polyadenylation sites and counting reads that map to either the distal or proximal PAS to define APA usage. Interestingly, spvAPA uses an imputation method to overcome sparseness and missing entries in the APA single-cell data (Zhang et al. 2025). Even though imputation is a common method to deal with dropouts in single-cell sequencing data, the balance between biological and technical sources of zeros should be taken into account. It can also induce statistical dependency between imputed observations, which should be considered for downstream analysis (Andrews and Hemberg 2018; Cheng et al. 2023).

#### Quantifying 3′UTRs from long-read isoform sequencing in single cells

Many obstacles remain as many isoforms share overlapping sequences, making it hard to be unambiguously mapped in short-read sequencing data especially if the upstream sequence is repetitive or similar to other isoforms. Therefore, an alternative approach to study 3′UTRs is to integrate long-read sequencing without the inherent 3′ bias to identify and map 3′UTR isoforms.

FLAM-seq leverages third-generation sequencing techniques, PacBio and ONT, and enables direct identification of full-length isoforms from “head to toe.” This includes transcription start sites, alternative splicing events, 3′UTR isoforms, and poly(A) tail lengths in bulk (Legnini et al. 2019). On a single cell scale, SCALPEL introduces a Nextflow-based pipeline for quantifying transcript isoforms and APA directly from 3′ scRNA-seq data using paired short- and long-read data sets. The authors reanalyzed developmental and differentiation data sets, and demonstrated that isoform and 3′UTR usage vary extensively across cell types. They thereby refined single-cell resolution of transcript diversity and could even show how miRNAs interact with 3′UTR shortening and lengthening through APA (Ake et al. 2025).

Overall, there has been substantial effort to analyze long-read sequencing data for isoform discovery. Existing computational tools can be broadly categorized into tools that combine single-cell or spatially barcoded short reads with long-read sequencing data to assign long reads to individual cells, such as ScISOr-Seq (Gupta et al. 2018), ScNapBar (Ren et al. 2023), SiCeLoRe (Lebrigand et al. 2020), FLAMES (Tian et al. 2021), Spl-ISO-Seq (Foord et al. 2025), and SiT (Spatial Isoform Transcriptomics) (Lebrigand et al. 2023); and tools that directly ingest long reads from ONT or PacBio sequencing, optionally with internal error-correction strategies for sequencing errors, such as IsoQuant (Prjibelski et al. 2023), ScISOr-Seq (Gupta et al. 2018), SiCeLoRe 2.1(Lebrigand et al. 2020), and Longcell (Fu et al. 2025). Although most of these studies do not explicitly focus on 3′UTRs, their use of long-read sequencing to capture full-length transcripts suggests potential applicability for 3′UTR isoform discovery. This is especially relevant in spatial context, which requires spatially barcoded single cells. Some of these approaches support spatial barcoding, while others indicate that spatial barcodes could be integrated in principle; others do not comment on this. Collectively, this underscores the promising opportunity for analyzing 3′UTR isoforms in a spatial context using long-read sequencing data.

Even though there is a full menu of available tools to tackle various challenges in short- and long-read sequencing with spatial resolution, the field still has some key experimental and computational bottlenecks that need to be addressed. Long-read sequencing offers important advantages for resolving full-length isoforms, yet it continues to struggle with higher error rates in comparison to short-read sequencing and transcript fragmentation during library preparation. On the computational site, these consist of the absence of standardized benchmarking frameworks, incomplete 3′UTR isoform annotation across cell types and tissues, and limited scalability of integrative models combining long-read and spatial transcriptomic data. Despite these challenges, rapid technological advancements and increasing availability of integrated methods promise to overcome these bottlenecks. Additionally, the curation of APA events and PolyAsites, for example scAPAdb (Zhu et al. 2022), PolyA_DB (Zhang 2004), and PolyASite v3.0 (Moon et al. 2025) helps to further expand the field. Combined with methods that allow to infer these events from 3′ sequencing data, also with spatially barcoded spots (e.g., stAPAminer, spvAPA), this is a valuable resource. Future work would consider integrating spatial data for posttranscriptional regulation events into databases to leverage the potential for understanding spatial patterns of 3′UTRs and opening exciting opportunities for deeper insights into spatial gene regulation.

### Revealing the interplay between miRNA and UTR isoforms: a road still under construction

Even though there are a lot of methods to investigate miRNAs and 3′UTR isoforms expression individually, it is important to note that miRNA-regulated gene expression largely relies on the abundance of miRNAs and the availability of accessible binding sites within their respective 3′UTR targets. Therefore, coprofiling miRNAs and isoforms is essential to move from correlation to understanding the regulatory mechanism, linking local miRNA abundance to isoform availability and subsequent downstream effects. The added advantage of single-cell and spatial resolution is required to distinguish region- and cell-specific mechanisms, which have already been hinted at through individual studies. Unfortunately, current methods rarely achieve high-throughput detection without compromising sensitivity, resulting in sparse data for miRNAs and isoforms. Additionally, 3′UTR isoform diversity collapses during analysis, leading to the loss of miRNA binding sites information and obscuring the true regulatory landscape.

#### Experimental investigation of miRNA–target interaction

The most common experimental approaches investigate miRNA–target interaction by crosslinking RNA–protein complexes, followed by immunoprecipitation of the AGO proteins and cDNA sequencing (Hafner et al. 2012). Several variants of this approach aim to improve its efficiency, for example by using tagged proteins (CLASH; Helwak and Tollervey 2014), by incorporating 4-thiouridine into cultured cells to enhance crosslinking efficiency (PAR-CLIP; Hafner et al. 2010), or by generating miRNA:mRNA chimeras by ligation (Grosswendt et al. 2014). Those methods allow for an explorative approach to analyze miRNA binding sites; however, the high amount of input material required for miRNA detection, complex workflows, and convoluted computational analysis have to be taken into consideration (for review, see Hafner et al. 2021). To resolve miRNA–mRNA interaction in space, Diag et al. performed mRNA and small RNA sequencing on cryo-cut slices of *C. elegans* and were able to study both mRNA and miRNA expression at near single-cell resolution. By mapping these spatiotemporally across the germline, they resolved spatial patterns, showing that alternative polyadenylation and 3′UTR isoform choice are highly zone-specific and integrated with miRNA activity (Diag et al. 2018).

#### Computational tools for miRNA–target interaction

In the early days of studying miRNA–mRNA interactions, scientists developed computational tools that focused on predicting miRNA target sites based on sequences. One of the first approaches for predicting miRNA target sites in a high-throughput manner, called PicTar, was presented by our laboratory in 2004 (Rajewsky and Socci 2004). Many more tools like TargetScan (Agarwal et al. 2015), miRanda (John et al. 2005), DIANA-microTv5.0 (Paraskevopoulou et al. 2013), and scanMiR (Soutschek et al. 2022) were developed. However, while these predictions clearly identified functional sites by focusing on evolutionarily conserved sequences, they suffered from false positive rates, for example, in situations where 3′UTR sequences are conserved for reasons beyond miRNA binding sites. These purely computational approaches therefore require orthogonal validations such as expression profiling coupled to CLIP-seq (Ule et al. 2003; Licatalosi et al. 2008). Recent advances using machine learning–based tools such as miRAW (Pla et al. 2018) and miTAR (Gu et al. 2021) try to overcome these limitations. These models typically ingest experimentally validated miRNA–mRNA interaction data sets or binding site annotations from CLIP-seq experiments, allowing better prediction outcomes. While miRAW uses full-length miRNA and 3′UTR sequences to learn interaction features through deep neural networks, miTAR applies a hybrid deep-learning model that learns both spatial and sequential features to identify potentially noncanonical binding sites.

Overall, these tools are able to predict miRNA–target interactions on a large scale with high accuracy and also integrate structural information (e.g., binding energy). However, they do not explicitly support data from spatial sequencing methods and are not able to integrate cell metadata yet (e.g., spatial localization or cell type information). Addressing this gap, miTEA-HiRes integrates single-cell and spatial transcriptomics data to predict and map miRNA activity in space. It leverages the miRTarBase (Hsu et al. 2011) database, filters miRNA candidates, and infers their activity from single-cell and spatial sequencing data. By doing this, miTEA-HiRes can predict activity on single-cell data that can be visualized on low-dimensional embeddings (e.g., UMAPs), or within spatial context or even between cell populations directly (Herbst et al. 2025). Similarly, STmiR is another tool that learns expression patterns from paired bulk miRNA–mRNA sequencing data and predicts miRNA activity on spot-level spatial transcriptomics data sets (Yuan et al. 2025). Despite their predictive performance, their integration of bulk and single-cell sequencing data, and their capability to map miRNA to function and cell types, these approaches still need to be used with caution. As miTEA-HiRes ingests the miRTarBase database, predictions would need additional biological validation (as mentioned above), and even though STmiR uses spatial transcriptomics data (Visium), they infer activity on spot-level, not in single cells and not explicitly to project those in space.

Taken together, the methods for detecting and analyzing miRNAs and 3′UTR isoforms have advanced considerably; in their ability to ingest data, in their resolution, and many more. While ISH-based methods remain crucial for high-resolution miRNA and isoform detection in space, they are limited by throughput. On the other hand, while sequencing-based methods provide this high-throughput of unbiased detection of both miRNAs and 3′UTR isoforms, it often comes at the cost of specificity and resolution. Moreover, truly integrated spatial sequencing approaches that quantify both modalities remain rare, limiting the field largely to inferring correlations between miRNA and their target interactions.

## FUTURE PERSPECTIVES

This review highlights how miRNAs, 3′UTRs, and their interplay orchestrate brain development and neuronal function. We also discuss how disruptions in either of these elements are linked to various neurodevelopmental and neurodegenerative disorders. The field is transitioning from supervised probe-based imaging to more unbiased spatial sequencing methods, evolving from merely quantifying gene expression to employing activity-aware models that account for 3′UTR isoforms and their local context. However, a significant challenge persists: We fail to simultaneously measure miRNAs and their respective 3′UTR isoforms within the same tissue, at native spatial (and subcellular) resolution. Addressing this gap should be the primary objective for the next wave of methodologies.

A promising direction is a dual-chemistry workflow that captures both small RNAs and 3′-end resolved mRNAs on the same slide, allowing for paired measurements of miRNA abundance and isoform usage. However, challenges such as competition for capture capacity and the presence of miRNAs bound to AGO proteins would need to be addressed. Alternative approaches involve spatial AGO-CLIP-profiling to capture miRNA–mRNA chimeras (Grosswendt et al. 2014) with adopted spatial transcriptomic protocols (Fig. 2C, left). However, such a method would need to integrate UV-crosslinking and chimeric-reads capture to existing spatial protocols, which is a feat by itself. The method would also need to address concerns including ligation efficiency, background noise, and unspecific polyadenylation. Nevertheless, a validation strategy focusing on well-characterized miRNA–target pairs could support the interactions obtained. Additionally, prediction tools might be promising in enhancing data resolution, but their usage requires caution, given the nature of their input.

Additionally, alignment-free alternatives such as *k*-mer-based strategies could tackle isomiR variation–related challenges. By skipping the step of mapping sequences to a reference genome, it could mitigate reference-dependent biases and give insights into biodiversity, because sequences could be directly compared across species (Fig. 2C, right). *k*-mers of sequences would be analyzed directly and could later be compared to genome databases.

These methods could help overcome current challenges in sensitivity and specificity, enabling more accurate capture of spatially resolved miRNA and 3′UTR interactions (Solomon and Kingsford 2016; Marchet et al. 2020).

Furthermore, implementing paired, subcellular coprofiling will help reconcile discrepancies from single-plex studies. Additionally, it can enhance the accuracy of causal inferences and clarify mechanisms in neurodegenerative diseases, where region-specific dysregulation of isoforms–miRNA pairs contributes to disease progression. Moreover, this advancement can create target-engagement maps for miRNA mimics/antagomiRs, reduce off-target risk by revealing cell-type-restricted expression, and yield biomarkers that outperform expression alone. Given the stability of miRNAs in biofluids (Pellegrini et al. 2022), utilizing miRNA-isoform spatial readouts can aid in better candidate selection, optimize dosing, and improve patient stratification. In the brain, an organ defined by complex spatial biology and cell-type diversity, such advances are required to bridge the gap between miRNA biology, biomarker detection, and therapeutic success.
